# Detection of African Swine Fever Virus from Wild Boar, Singapore, 2023

**DOI:** 10.3201/eid2912.230966

**Published:** 2023-12

**Authors:** Eileen Y. Koh, Adrian K.S. Tan, Darren Yeo, Clara Lau, Li Ying Tan, Oi Wing Ng, Jasmine Ong, Stacy Chong, Steffie Toh, Jing Chen, Wai Kwan Wong, Brian Z.Y. Tan, Christine He-Lee, Zhan Pei Heng, Ian Liang, Charlene Judith Fernandez, Siow Foong Chang, Kenneth B.H. Er

**Affiliations:** National Parks Board, Singapore

**Keywords:** African swine fever, ASFV, viruses, vector-borne infections, parasites, wild boar, swine diseases, Singapore

## Abstract

We detected African swine fever virus (ASFV) from a wild boar in Singapore. In <72 hours, we confirmed and reported ASFV p72 genotype II, CD2v serogroup 8, and IGR-II variant by using a combination of real-time PCR and whole-genome sequencing. Continued biosurveillance will be needed to monitor ASFV in Singapore.

African swine fever (ASF), a nonzoonotic, World Organisation for Animal Health (WOAH) notifiable disease, is a devasting hemorrhagic infectious disease of domestic (*Sus domesticus*) and wild (*Sus scrofa*) swine populations (*1*). ASF was identified in Kenya in 1921 and subsequently spread through >50 countries (*1*). ASF is known to be spread by *Ornithodorus* soft ticks, vectors of ASF virus (ASFV), as well as contact with infected swine or contaminated vehicles or equipment and by consuming of infected carcasses (*1*). ASF often is associated with high illness and death rates in suids. However, wild suid species in Africa, such as warthogs (*Phacohoerus aethiopicus*), act as reservoir hosts for the virus but reportedly remain asymptomatic (*1*). The occurrence of ASF in affected countries has caused substantial economic losses in the swine industry, amounting to billions of US dollars globally (*2*).

ASF is caused by a large, enveloped DNA virus ≈200 nm in diameter, and ASFV is the only member of the family *Asfarviridae*. The ASFV genome contains 170–193 kb of double-stranded DNA (*1*); the observed range in genome size is primarily due to gain or loss of gene copies belonging to multigene families and variation within the number of tandem repeats in noncoding regions of the ASFV genome (*3*).

The absence of a safe and effective vaccine against ASFV and limited information on the spatial distribution of wild boar in Asia has restricted effective disease control measures and outbreak management (*4*). We report detection of ASFV in a wild boar in Singapore.

A wild boar (*S. scrofa*) carcass was found in the northwestern part of the Singapore main island on February 5, 2023, and was submitted to the Centre for Animal and Veterinary Sciences for disease investigation. The extensive spread of ASF within the region (*5*,*6*), coupled with the necropsy findings of hemothorax, hemoperitoneum, and widespread subcutaneous and pulmonary hemorrhage within the carcass, prompted us to include ASF as one of the key differential diagnoses. 

We obtained samples from 7 organs (liver, lung, heart, spleen, lymph nodes, kidney, and tonsil) and 2 fluid samples (abdominal and thoracic fluids) from the carcass for virological analysis. In addition, we collected 2 adult ticks (1 male and 1 female) from the carcass and identified them as *Dermacentor auratus* ticks by DNA barcoding (*7*). As part of the disease investigation, we removed the residual host tissue from the ticks’ mouthparts before using the whole tick for nucleic acid extraction using the DNeasy Blood and Tissue Kit (QIAGEN, https://www.qiagen.com).

We extracted viral DNA from the 7 organ and 2 fluid samples by using the IndiMag Pathogen Kit (Indical Bioscience GmbH, https://www.indical.com), according to the manufacturer’s guidelines. We detected ASFV from the extracted nucleic acids from all 11 (9 suid and 2 tick) samples by real-time PCR (*1*); cycle threshold values were 19.82–33.83.

We constructed an ASFV-positive library by using the LSK109 Ligation Sequencing Kit (Oxford Nanopore Technologies, https://nanoporetech.com) and Illumina DNA Prep Kit (Illumina, https://www.illumina.com). Then we performed whole-genome shotgun sequencing by using an R9.4.1 flow cell on the MinION (Oxford Nanopore Technologies) platform and the iSeq 100 (Illumina) platform, according to the manufacturers’ specifications. We were able to retrieve full-length sequences from the wild boar samples but not from the tick samples. The overall sequence similarity from both platforms, based on an ungapped alignment to the reference ASFV sequence Georgia 2007/1 (GenBank accession no. FR682468.2), was 99.89%. We used SAMtools consensus version 1.17 and the default Bayesian counting (*8*) to merge the final ASFV sequence from both sequencing technology platforms. The merged sequence had 99.57% coverage and a mean depth of 14.49. We deposited the full-length ASFV genome (190,148 nt) from this study into GenBank (accession no. OR135685).

Genotyping of the p72 gene and serotyping with 90 nt from the EP402R gene have been used to characterize ASFV strains to provide possible viral origins and differentiation between closely related strains (*9*). The Singapore ASFV strain was classified as genotype II, based on monophyly (Figure, panel A), and serogroup 8 (Figure, panel B). Compared with the reference sequence, FR682468.2, the Singapore strain also showed insertion of an additional 10-bp tandem repeat sequence (5′-GGAATATATA-3′) between the intergenic region of the I73R and I329L gene (*10*), which is consistent with ASFV sequences reported in the region as IGR-II variant (*5*,*6*).

**Figure:**
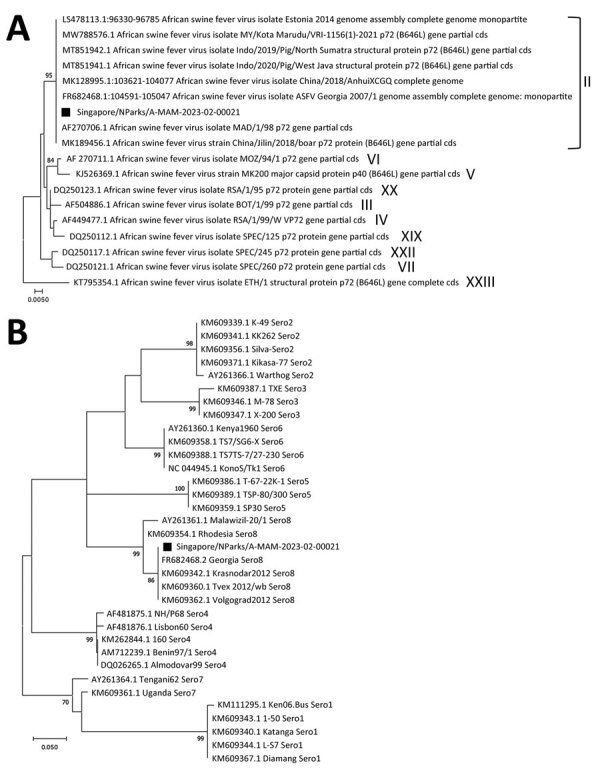
Phylogenetic analysis of African swine fever virus detected in a wild boar, Singapore, 2023. A) Analysis of p72 genotype. Roman numerals to the right indicate the respective genotypes; 10 of 24 known genotypes are shown. B) Analysis of CD2v serogroups constructed by using the maximum-likelihood method and Tamura-Nei model with 1,000 bootstrap values in MEGA X software (https://www.megasoftware.net). Only bootstrap values >70% are shown. Black squares indicate sample from this study (Singapore/NParks/A-MAM-2023-02-00021; GenBank accession number OR135685). GenBank accession numbers are provided for all reference sequences. Scale bars indicate nucleotide substitutions per site.

The combination of real-time PCR and high-throughput sequencing enabled rapid confirmation of the ASFV in Singapore within 72 hours of detection of the index carcass. We subsequently notified ASFV detection to WOAH on February 7, 2023, and Singapore initiated islandwide ASF control and management measures. 

In conclusion, we detected ASFV in a wild boar in Singapore. How and when the virus was introduced into the local wild boar population and the significance of the *D. auratus* tick in ASFV transmission in Singapore remain to be determined. Further studies are ongoing to elucidate the effects of this ASF incursion to the local wild boar populations. Continued biosurveillance will be needed to monitor ASFV in swine in Singapore. 
